# Prognostic significance and therapeutic potential of the immune checkpoint VISTA in pancreatic cancer

**DOI:** 10.1007/s00432-020-03463-9

**Published:** 2020-11-25

**Authors:** Zelin Hou, Yu Pan, Qinglin Fei, Yali Lin, Yuanyuan Zhou, Ying Liu, Hongdan Guan, Xunbin Yu, Xianchao Lin, Fengchun Lu, Heguang Huang

**Affiliations:** 1 Department of General Surgery, Fujian Medical University Union Hospital, No. 29 Xinquan Road, Fuzhou, 350001 People’s Republic of China; 2 The Cancer Center, Fujian Medical University Union Hospital, Fuzhou, 350001 China; 3 Department of Geriatrics, Fujian Medical University Union Hospital, Fuzhou, 350001 China; 4 Department of Endocrinology, Quanzhou Hospital of Traditional Chinese Medicine, Quanzhou, 362000 China; 5 Department of Radiation Oncology, Fujian Medical University Union Hospital, Fuzhou, 350001 China; 6 Department of Pathology, Fujian Provincialial Hospital, Fuzhou, 350001 China

**Keywords:** VISTA, Immune checkpoints, Immunotherapy, Pancreatic cancer, Biomarker

## Abstract

**Objective:**

V-domain Ig suppressor of T cell activation (VISTA) is a novel immune checkpoint protein that belongs to the B7 family. The aim of this study was to investigate the prognostic significance and therapeutic potential of VISTA in patients with pancreatic cancer.

**Methods:**

Using immunohistochemistry (IHC), we examined the expression of VISTA and demonstrated the associations between the VISTA and overall survival in 223 PDAC patients from 2 different unrelated retrospective cohorts. The multiplex immunofluorescence was performed to illuminate the relationship between VISTA expression and tumor-infiltrating immune cell subclusters of PDAC. We also verified the findings in The Cancer Genome Atlas (TCGA) dataset. The anti-tumor effect of anti-VISTA therapy was studied by the mouse model with liver metastases of PDAC.

**Results:**

The VISTA protein was highly expressed in 25.6% of tumor cells (TCs), 38.1% of immune cells, and 26.0% of endothelial cells in 223 PDAC tumor tissues. VISTA expression in TCs was significantly associated with prolonged overall survival. Multiplex immunofluorescence analysis revealed that VISTA level was positively correlated with CD68^+^ macrophages, CD3^+^ T cells, and CD19^+^ B cells in PDAC. However, a higher expression level of VISTA was detected in tumor-infiltrating CD68^+^ macrophages than in CD3^+^ T and CD19^+^ B cells. Furthermore, anti-VISTA antibody treatment significantly reduced the number of metastatic nodules in livers of mouse models of PDAC with liver metastases.

**Conclusion:**

VISTA expressed in TCs is associated with a favorable prognosis in PDAC. Moreover, immunotherapy with anti-VISTA antibodies may potentially be an effective treatment strategy against PDAC.

**Electronic Supplementary Material:**

The online version of this article (10.1007/s00432-020-03463-9) contains supplementary material, which is available to authorized users.

## Introduction

Pancreatic ductal adenocarcinoma (PDAC) is the most lethal type of human cancer, with a low 5-year survival rate of 6–10% (Yao et al. 2020). Recently, although programmed cell death protein 1 (PD-1)/programmed cell death ligand 1 (PD-L1) blocking therapy has been used successfully in several types of malignancies (Brahmer et al. 2012; Hagi et al. 2020; Rao et al. 2020; Tomita et al. 2019), the response rate is low in PDAC patients (Brahmer et al. 2012; Tomita et al. 2019; Wainberg et al. 2020). Thus, it is imperative to investigate the mechanism of immune escape in PDAC and potential alternate therapeutic strategies.

Previous studies indicated that tumor-associated macrophages (TAMs) were abundant in the tumor microenvironment (TME) of PDAC (Chen et al. 2018; Habtezion et al. 2016; Zhu et al. 2017). Therefore, TAMs may be a useful therapeutic target against PDAC. CD47 is a signaling molecule expressed on malignant cells to suppress macrophage phagocytosis. In our previous study, we found that anti-CD47 therapy was effective in mouse models of PDAC (Pan et al. 2019b). Moreover, Blando et al. (2019) found that V-domain Ig suppressor of T cell activation (VISTA) was an immune checkpoint that was highly expressed in macrophages of PDAC, and revealed that VISTA may be a potential therapeutic target for this disease.

VISTA, also known as VSIR, B7-H5, PD-1H, and SISP1, belongs to the B7 family of immune checkpoint proteins, that is homologous with PD-L1 (Ni and Dong 2017; Wang et al. 2011). VISTA is mainly expressed in tumor-infiltrating immune cells (ICs) within the TME. However, in recent studies, VISTA was also detected in membranes of tumor cells (TCs), such as gastric, ovarian, and lung cancers (Boger et al. 2017; Mulati et al. 2019; Villarroel-Espindola et al. 2018; Zong et al. 2020b). VISTA expresses on T cells as a part of an inhibitory immune checkpoint that suppresses T cell activation, proliferation, and cytokine production (ElTanbouly et al. 2020; Flies et al. 2011; Lines et al. 2014). However, the biological function of VISTA is still unclear in pancreatic cancer. Specifically, the expression of VISTA in PDAC tumor tissues has not been studied comprehensively. In addition, the correlation between VISTA expression and immune cells infiltration within the TME remains uninvestigated. Finally, the anti-tumor effect of VISTA targeting therapy in PDAC has not been verified.

In this study, we performed immunohistochemistry (IHC) and multiplex quantitative immunofluorescence in two independent cohorts, and then verified the findings in The Cancer Genome Atlas (TCGA) dataset. We found that VISTA was expressed in TCs, ICs, and endothelial cells (ECs) in the TME of PDAC, and was associated with PD-1/PD-L1 expression as well as clinical outcomes of PDAC patients. Furthermore, we demonstrated that anti-VISTA antibody treatment significantly suppressed the progression of liver metastatic tumors in mouse models of PDAC. These findings implicate VISTA as an effective prognostic factor and novel potential immunotherapeutic target for PDAC.

## Materials and methods

### Patients and tissue samples

The formalin-fixed, paraffin-embedded tumor samples from 223 stage I—IV PDAC patients in two cohorts were retrospectively collected. The first cohort is from Fujian Medical University Union Hospital between December 2010 and December 2018 and included 137 cases. The second cohort is from Fujian provincial hospital and included 86 cases collected between September 2005 and December 2012. All cases were verified as PDAC by histological examination. And all samples were enrolled in this study after excluding patients with neoadjuvant treatment, inflammatory diseases or active infection. The following patient characteristics were collected and evaluated. The stage of each patient was assessed based on the American Joint Committee on Cancer version 8 (AJCC 8). Informed consent had been obtained previously. The study was approved by the Committee for the Ethical Review of Research, Fujian Medical University Union Hospital.

Construction of TMAs was performed by Shanghai Outdo Biotech Company, Shanghai. In brief, a hollow needle is used to remove tissue cores as small as 1.5 mm in diameter from paraffin-embedded tumor tissues of the cohort #1. Three spots was punched out of the tumor from each cases and TMA cores with tumor and stromal contents < 5% were excluded. And the tissue cores were inserted in a paraffin block to establish an array pattern. Sections were cut from the array pattern for further study.

### Immunohistochemistry (IHC)

All IHC experiments were performed in the large tissue sections, which cut from paraffin-embedded samples. Protocol of IHC was described previously (Pan et al. 2019a). The sections were deparaffinized and pretreated in 1 mmol/L EDTA at 95 ℃ 20 min for antigen retrieval, then incubated with 0.3% hydrogen peroxide solution for 15 min for block endogenous peroxidase activity. Primary antibodies were used to detect VISTA (CST, Clone D5L5T; 1:25) and PD-L1 (Abcam, Clone EPR19759; 1:250). For visualization, Elivision super HRP (Mouse/Rabbit) IHC Kit (KIT-9922) and DAB Kit (20×) (DAB-0031) were used follow the instructions. Counterstaining was done with hematoxylin. PD-L1 was detected in the cytoplasm/membrane of TCs, whereas VISTA was detected in the cytoplasm/membrane of TCs, ICs, and ECs.

IHC analysis of PD-L1 status were performed in our previous study (Pan et al. 2019a). VISTA expression was evaluated in TCs, ICs and ECs, respectively. Evaluation of VISTA expression in TCs and ICs were referred to previously published articles (Boger et al. 2017; Schoop et al. 2020). The VISTA expression in TCs were defined based on the intensity score as 0 for negative, 1 for weak, 2 for moderate, or 3 for strong, and the percentage of TCs stained with VISTA positive was assessed based on the score of 1–3 representing  < 10%, 10–30%, and  > 30% cells. The final score of each sample was calculated by adding the scores for the staining intensity and the percentage of VISTA‑positive cells. TCs were considered as VISTA high expression if the score is  ≥ 3. Ten areas of a representative field were counted at ×400 magnification for VISTA-positive ICs, and the average was calculated. High infiltration of VISTA^+^ ICs was defined as more than 200 positive cells on average. ECs were accessed as VISTA-high expression if any staining was present excess 5% of these cells, as described in previous study (Zong et al. 2020a, b). All specimens were evaluated by two pathologists who were blinded to the patients’ clinical information.

### Multiplex immunofluorescence

Multiplex immunofluorescence was performed using a previous protocol (Gorris et al. 2018). Sections were cut from the TMAs and control tonsil tissue, and were deparaffinized, rehydrated, and then treated with EDTA buffer. 3% hydrogen peroxidase was used to block the endogenous peroxidase. Slides were incubated with the primary CD3 (Abcam, Clone PS1; 1:100), CD4 (Abcam, Clone EPR6855; 1:100), CD8 (Abcam, Clone EP1150Y; 1:500), CD19 (Abcam, Clone EPR5906; 1:500), CD68 (CST, Clone D4B9C; 1:10,000), PD-1 (Abcam, Clone NAT105; 1:25), PD-L1 (Abcam, Clone EPR19759; 1:250), VISTA (CST, Clone D5L5T; 1:25), and pan-CK (Abcam, Clone PAN-CK (Cocktail); 1:800) antibodies for 1 h at room temperature. Then, incubation with BrightVision poly-HRP-anti-Ms/Rb/Rt IgG (DPVO999HRP; Immuno-Logic) at room temperature for 10 min. Opal reagent (The Opal 7-color IHC Kit; NEL797B001KT; 1:100) for 10 min at room temperature was performed for color development. Next, epitope retrieval was performed by microwave treatment to remove the antibody complex. After dehydration, slides were counterstained with DAPI for 5 min and were enclosed with VECTASHIELD HardSet Antifade Mounting Medium (H-1400; Vector Labs). The experimental conditions and multiplex immunofluorescence panels are summarized in Supplementary Table S1.

Using each individually stained section for each reagent to establish the spectral libraries and separating the multispectral images by the inform Advanced Image Analysis software (inForm 2.4.2; PerkinElmer). Slides were scanned using the PerkinElmer Vectra slide scanner (Vectra 2.0.7 and 3.0.3; PerkinElmer). The inForm software was used to calculate the positive score. The colocalization of PD-1/PD-L1 and VISTA staining was estimated using the colocalization algorithm. The analysis was performed as previously described (Gao et al. 2017; Stack et al. 2014).

### Liver metastases models

Panc02 or MPC-83 cells (1 × 10^6^ cells in 50 μl PBS) were injected into the spleens of C57BL/6 or KM mice. Two weeks after the injections, the mice were randomly divided into two groups for seven per set, and treated with anti-VISTA monoclonal antibody (mAb; 200 μg/day i.p., Clone No. 13F3, BioXcell) or mouse IgG (200 μg/day i.p., Clone No. MPC-11, BioXcell). Three weeks after treatment, mice were sacrificed under deep anesthesia and the liver were harvested. Tumor metastases were quantified by counting the number of metastatic colonies on one histological section at the middle portion of each liver sample.

### Cell lines and mice

The murine PDAC cell lines Panc02 and MPC-83 were obtained from Shanghai Aolu Biological Technology Co. Ltd (Shanghai, China). All cell lines were tested to rule out mycoplasma contamination.

Male C57BL/6 mice, 4 weeks of age, and male Kunming (KM) mice, 4–5 weeks of age, were obtained from Beijing Vital River Laboratory Animal Technology Co., Ltd. (Beijing, China). Animal study was performed at the Comparative Medicine Center of 900 Hospital of the Joint Logistics Team. Animal experiment protocols were approved by the Ethics Committee for Animal Research of 900 Hospital of the Joint Logistics Team.

### Statistical analyses

Quantitative data are expressed as the mean ± standard deviation and analyzed based on variance and Student’s *t* tests. χ^2^ tests were performed to compare VISTA/PD-L1 expression and clinical features. Spearman’s rank correlation was evaluated to determine the correlation between VISTA and PD-L1 expression. The differences in immunostaining among each group were analyzed by the one-way ANOVA followed by the Bonferroni multiple comparison tests. Overall survival was measured from the date of diagnosis to the day of death from any cause or the last censored follow-up. Survival analysis methods were described in our previous study (Pan et al. 2019a). Statistical significance was defined as a *p* value < 0.05. SPSS Statistics (Version 23, SPSS) was used for statistical analysis.

### TCGA data analysis

To analyze the PDAC samples from the TCGA (https://tcga-data.nci.nih.gov), we downloaded the RNA sequencing data from 177 PDAC cases. Then, the data were TPM-normalized and ENSG-ID transformed. Then, we evaluated the mRNA expressions of VISTA, CD68, CD19, CD3, CD4, CD8, PD-L1, and PD-1.

## Results

### Patient characteristics

The clinicopathological characteristics of 137 PDAC patients from cohort #1 and 86 PDAC patients from cohort #2 are summarized in Supplementary Table S2. In these two cohorts, the median age of patients were 62 years (35–80) and 62 years (34–83), with 35.8 and 56.4% females, and most had moderately differentiated (102 and 51 cases) or poorly differentiated (28 and 29 cases) grading. Neoadjuvant therapy was not performed in any patients of both two cohorts. Median overall survival was 12.0 and 8.0 months.

### VISTA expression in PDAC

**Fig. 1:**
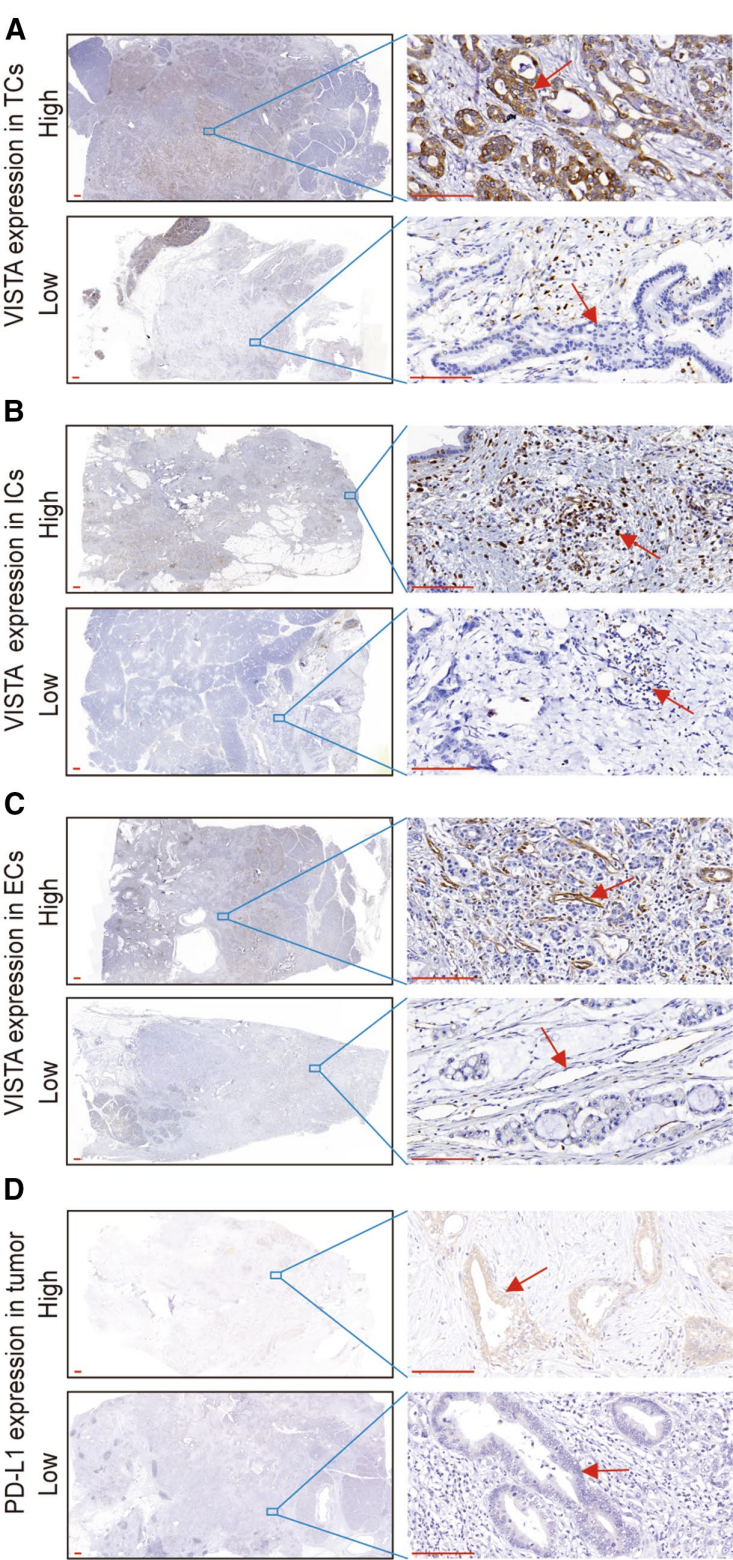
Immunohistochemical staining of VISTA and PD-L1 in human PDAC. Human PDAC tissue samples were stained with anti-VISTA and anti-PD-L1 antibodies. Low magnification (10×) and high magnification (400×) images were obtained. Scale bar = 50 μm (red line at the bottom left). **a** VISTA expression in tumor cells (TCs). The red arrows indicate VISTA-positive or VISTA-negative TCs. **b** VISTA expression in immune cells (ICs). The red arrows indicate VISTA-positive or VISTA-negative ICs. **c** VISTA expression in endothelial cells (ECs). The red arrows indicate VISTA-positive or VISTA-negative ECs. **d** PD-L1 expression in TCs. The red arrows indicate PD-L1-positive or PD-L1-negative TCs

Previous studies indicated that VISTA was detected frequently in the TME of several solid tumors (Deng et al. 2016; He et al. 2020; Liao et al. 2018; Rosenbaum et al. 2020). In this study, we extensively explored the expression of VISTA in 223 PDAC tumor tissues by IHC staining of each large section. The VISTA protein was detected in 99% (221/223) of all cases, and was found in TCs, ICs, and ECs. Representative IHC photomicrographs of VISTA are shown in Fig. 1.

In TCs, the percentage of positively stained cells varied from 0 to 80%, and the staining intensity ranged from weak to strong. By using the histological score (see Methods), we defined VISTA high expression in TCs as a score ≥ 3. A total of 26.3% (36/137) and 24.4% (21/86) of cases in cohort #1 and cohort #2 showed high expression of VISTA in TCs, respectively (Fig. 1a and Supplementary Table S3). This indicated that the overall number of VISTA-high TCs was low.

In ICs, the number that were VISTA-positive per field (400×) varied from 13 to 589 (median 168). Patients with less than 200 VISTA-positive ICs were classified as VISTA-low in ICs, while patients with  > 200 VISTA-positive ICs were defined as VISTA-high in ICs. We found that 40.2% (55/137) and 34.9% (30/86) of cases in cohort #1 and cohort #2 were VISTA-high in ICs (Fig. 1b and Supplementary Table S3). These data revealed that tumor-infiltrating ICs showed strong expression of VISTA.

In ECs, 23.4% (32/137) and 30.2% (26/86) of cases in cohort #1 and cohort #2 were classified as VISTA-high, which was defined as positive staining in excess of 5% of all ECs (Fig. 1c and Supplementary Table S3). Altogether, these results demonstrated that VISTA was expressed comprehensively in tumor tissues of PDAC, especially in tumor-infiltrating ICs.

Notably, the expression levels of VISTA in TCs and ECs were positively correlated in both cohort #1 and cohort #2 (*r* = 0.219, *p* = 0.01; *r* = 0.333, *p* = 0.002; Supplementary Table S4), whereas the level of VISTA in ICs did not correlate with VISTA expression in TCs or ECs (Supplementary Table S4). Moreover, the levels of VISTA were barely expressed in the adjacent normal pancreas tissues (Supplementary Fig S1).

### Prognostic significance

**Fig. 2:**
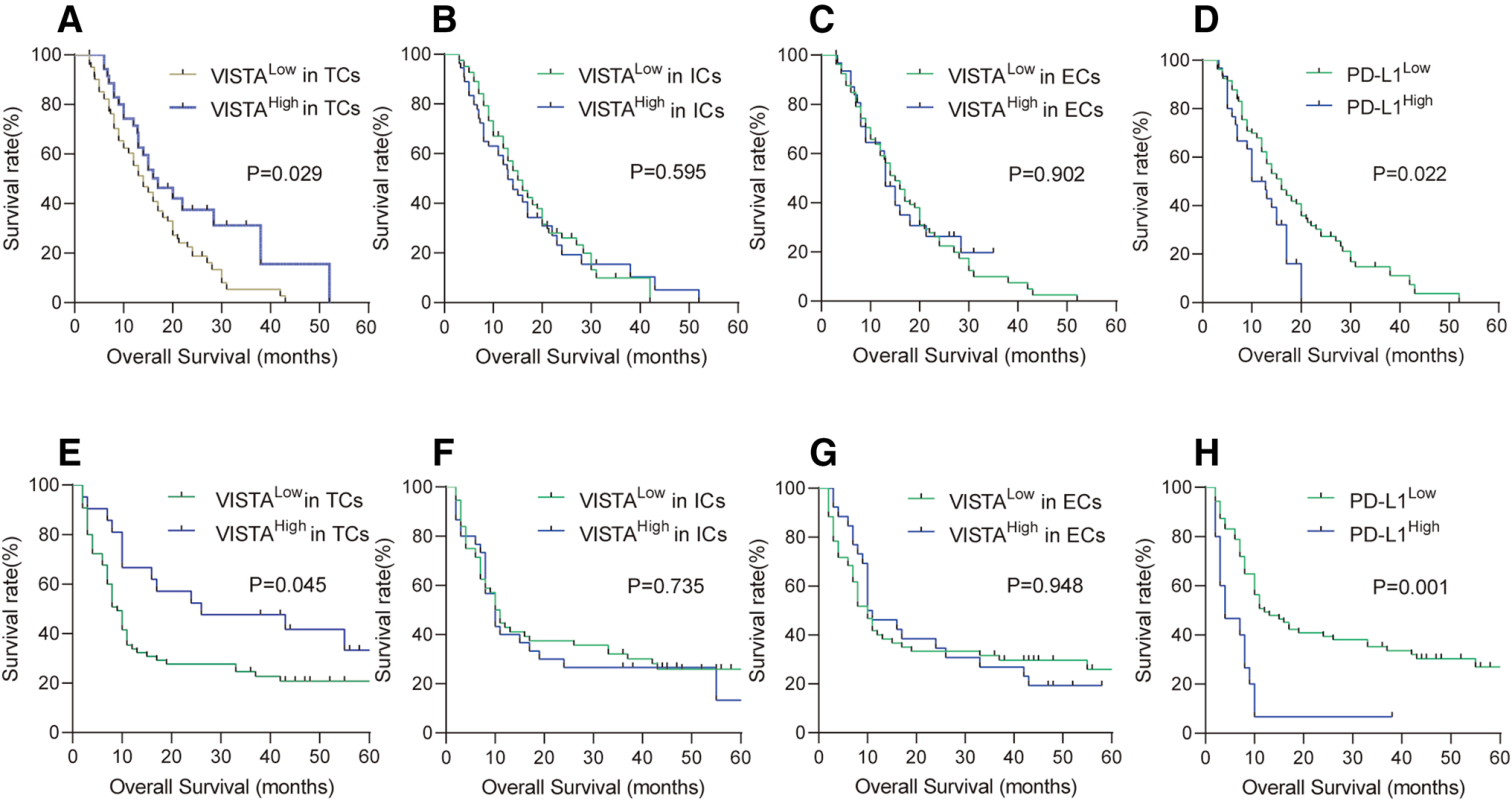
Kaplan–Meier analysis of OS of PDAC patients from cohort #1(**a–d**) and cohort #2(**e–h**) according to VISTA and PD-L1 expressions. (**a, e**) Kaplan–Meier plot of OS in cohort #1 and cohort #2 according to VISTA expression in TCs. (**b, f**) Kaplan–Meier plot of OS in cohort #1 and cohort #2 according to VISTA expression in ICs. (**c, g**) Kaplan–Meier plot of OS in cohort #1 and cohort #2 according to VISTA expression in ECs. (**d, h**) Kaplan–Meier plot of OS in cohort #1 and cohort #2 with high or low tumor expression of PD-L1

To explore the impact of VISTA and PD-L1 expression on clinical outcomes of PDAC patients, the Cox proportional hazards regression was performed. In cohort #1 (*N* = 137), univariate analysis showed that the variables associated with overall survival included grading [hazard ratio (HR) = 3.911, *p* = 0.012], tumor localization (HR = 0.599, *p* = 0.016), TNM stage (HR = 2.885, *p* = 0.006; HR = 4.221, *p* = 0.001; HR = 5.57, *p* = 0.001), and postoperative chemotherapy (HR = 0.488, *p* = 0.001; Table 1). Moreover, high expression of VISTA in TCs was significantly associated with a favorable overall survival (HR = 0.588, *p* = 0.029; Table 1; Fig. 2a); however, VISTA expression in ICs and ECs was not associated with survival (Table 1; Fig. 2b, c). The overall survival of patients whose TCs exhibited a low expression of PD-L1 was significantly longer than that of patients who exhibited a high expression of PD-L1 (HR = 1.759, *p* = 0.022; Table 1; Fig. 2d). Furthermore, a multivariate analysis was performed to investigate if VISTA and PD-L1 expression remained independent predictors for overall survival. VISTA expression in TCs, tumor expression of PD-L1, tumor localization, grading, TNM stage, and postoperative chemotherapy were incorporated into the multivariate analysis. The results showed that VISTA expression in TCs (HR = 0.452, *p* = 0.003), PD-L1 (HR = 2.242, *p* = 0.002), tumor localization (HR = 0.507, *p* = 0.002), grading (HR = 2.661,6.182; *p* = 0.081, 0.002), TNM stage (HR = 2.858, 4.062, 6.93; *p* = 0.007,  < 0.001,  < 0.001), and postoperative chemotherapy (HR = 0.474, *p* = 0.001, Table 1) were independent factors for prognosis.

**Table 1:** Univariate and multivariate Cox proportional analysis for overall survival (cohort #1, *N* = 137)

	*n*	Univariate analysis			Multivariate analysis		
		HR	95% CI	*p* value	HR	95% CI	*p *value
Gender							
Male	88	1					
Female	49	0.884	0.585–1.336	0.558			
Age (years)							
≥ 65	57	1					
< 65	80	0.855	0.573–1.276	0.444			
Location							
Head	88	1			1		
Other	49	0.599	0.394-0.911	0.016	0.507	0.327–.786	0.002
Grading							
G1	6	1			1		
G2	102	1.728	0.628–4.755	0.29	2.661	0.887–7.981	0.081
G3	28	3.911	1.352–11.311	0.012	6.182	1.998–19.128	0.002
G4^a^	1	–	–	–	–	–	–
pStage							
I	21	1			1		
II	63	2.885	1.354–6.144	0.006	2.858	1.331–6.137	0.007
III	45	4.221	1.977–9.014	< 0.001	4.062	1.881–8.771	< 0.001
IV	8	5.57	2.075–14.95	0.001	6.93	2.357–20.38	< 0.001
Vascular invasion							
Yes	37	1					
No	100	1.027	0.66–1.596	0.907			
CA199 (U/ml)							
< 37	33	1					
≥ 37	104	1.486	0.928–2.378	0.099			
Diameter (cm)							
≥ 4	69	1					
< 4	68	1.477	1–0.182	0.0503			
Procedure							
PD	85	1					
DP	43	0.688	0.446–1.061	0.09			
TP	9	0.747	0.323–1.728	0.495			
Postoperative chemotherapy							
No	57	1			1		
Yes	80	0.488	0.324–0.734	0.001	0.474	0.312–0.722	0.001
VISTA in TCs							
Low	101	1			1		
High	36	0.588	0.365–0.946	0.029	0.452	0.269–0.757	0.003
VISTA in ICs							
Low	82	1					
High	55	1.116	0.745–1.671	0.595			
VISTA in ECs							
Low	105	1					
High	32	0.97	0.603–1.561	0.902			
PD-L1							
Low	107	1			1		
High	30	1.759	1.085–2.853	0.022	2.242	1.344–3.738	0.002

*HR* hazard ratio, *CI* confidence interval, *TC* tumor cells, *IC* immune cells, *ECs* endothelial cells, *PD* pancreaticoduodenectomy, *DP* distal pancreatectomy, *TP* total pancreatectomy

^a^Because small number of cases in these groups, the survival analysis was meaningless

We further confirmed the prognostic power of VISTA on cohort #2 including 86 cases. Univariate analysis indicated that the grading, TNM stage, and postoperative chemotherapy were associated with overall survival (Table 2). Patients of VISTA high expression in TCs had long-term survivors (HR = 0.535, *p* = 0.045; Table 2; Fig. 2e), whereas VISTA expression in ICs and ECs were not correlated with survival (Table 2; Fig. 2f, g), which were similar to the results in cohort #1. PD-L1 high-expression tumors represent a short-term survivor (HR = 2.951, *p* = 0.001; Table 2; Fig. 2h). Moreover, multivariate analysis also showed that VISTA expression in TCs (HR = 0.481, *p* = 0.033), PD-L1 (HR = 3.503, *p* < 0.001), grading (HR = 2.187; *p* = 0.006), and TNM stage (HR = 3.119, 3.501, 3.585; *p* = 0.036, 0.023, 0.062; Table 2) remain prognostic markers.

**Table 2:** Univariate and multivariate Cox proportional analysis for overall survival (cohort #2, *N* = 86)

	*n*	Univariate analysis		Multivariate analysis			
		HR	95% CI	*P* value	HR	95% CI	*P *value
Gender							
Male	55	1					
Female	31	0.819	0.488–1.374	0.448			
Age (years)							
≥ 65	36	1					
< 65	50	1.241	0.755–2.040	0.396			
Location							
Head	54	1					
Other	32	1.155	0.701–1.903	0.571			
Grading							
G1^a^	3	–	–	–	–	–	–
G2	51	1			1		
G3	29	2.139	1.272–3.596	**0.004**	2.187	1.256–3.808	**0.006**
G4^a^	3	–	–	–	–	–	–
pStage							
I	11	1			1		
II	38	4.037	1.416–11.507	**0.009**	3.119	1.075–9.047	**0.036**
III	30	3.694	1.274–10.717	**0.016**	3.501	1.184–10.353	**0.023**
IV	7	5.452	1.524–19.497	**0.009**	3.585	0.938–13.706	0.062
Vascular invasion							
Yes	25	1					
No	61	1.349	0.805–2.262	0.256			
Diameter (cm)							
≥ 4	26	1					
< 4	60	0.836	0.498–1.402	0.497			
Procedure							
PD	50	1					
DP	27	1.149	0.670–1.969	0.613			
TP	9	1.149	0.484–2.728	0.752			
Postoperative chemotherapy							
No	47	1	0.270–0.750			0.329–1.022	
Yes	39	0.450		**0.002**	0.580		0.06
VISTA in TCs							
Low	65	1			1		
High	21	0.535	0.290–0.987	**0.045**	0.481	0.246–0.943	**0.033**
VISTA in ICs							
Low	56	1					
High	30	1.092	0.655–1.822	0.735			
VISTA in ECs							
Low	60	1					
High	26	0.983	0.582–1.658	0.948			
PD-L1							
Low	71	1			1		
High	15	2.951	1.592–5.469	**0.001**	3.503	1.799–6.821	**< 0.001**

*HR* hazard ratio, *CI* confidence interval, *TC* tumor cells, *IC* immune cells, *ECs* endothelial cells, *PD* pancreaticoduodenectomy, *DP* distal pancreatectomy, *TP* total pancreatectomy

^a^Because small number of cases in these groups, the survival analysis was meaningless

### VISTA expression and the tumor immune microenvironment

**Fig. 3:**
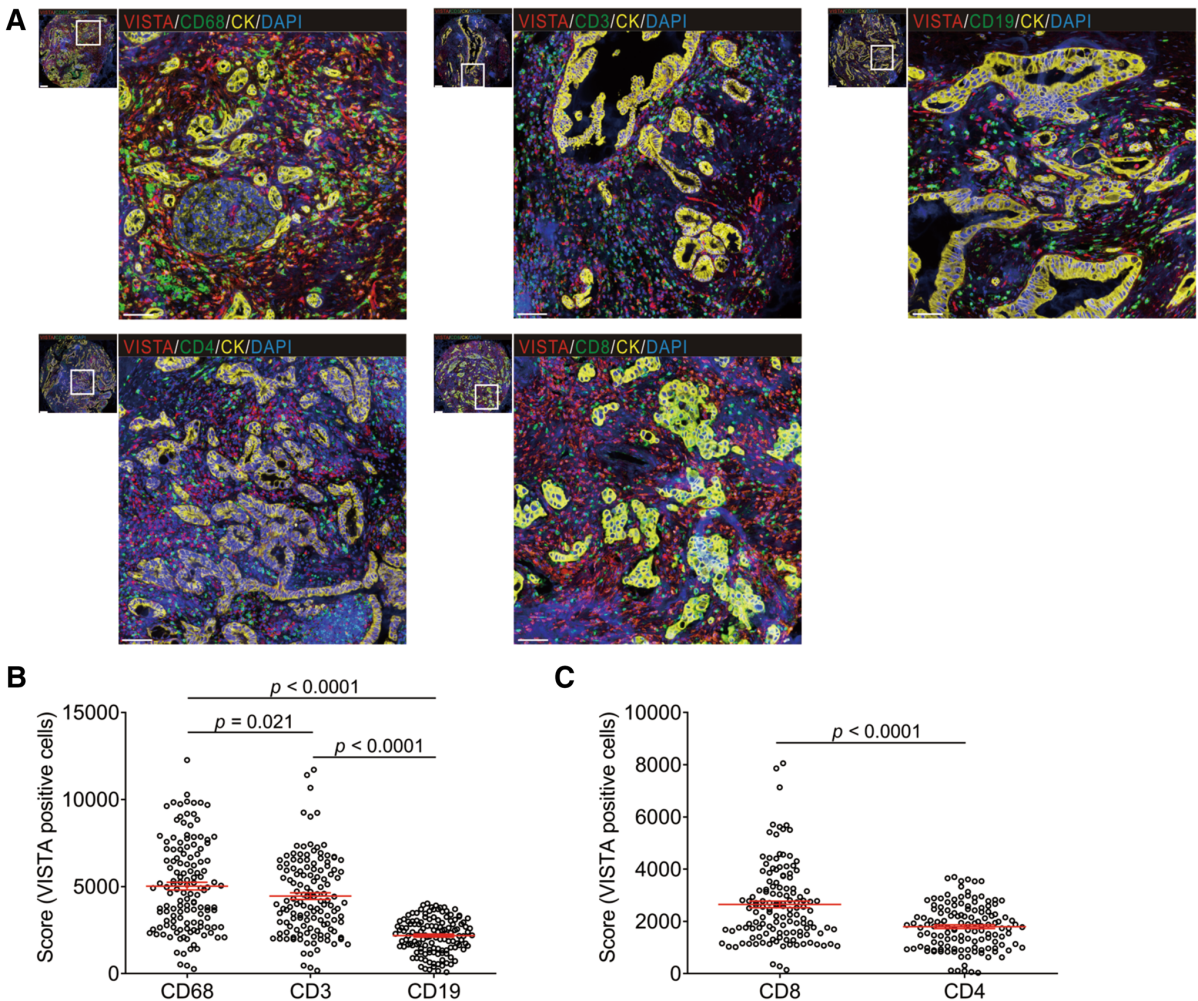
VISTA expression in immune cells subsets. **a** Representative images of coexpression of VISTA, immune cell markers (CD68^+^, CD3^+^, CD19^+^, CD4^+^, or CD8^+^ cells), and tumor cells (CK^+^ cells). **b** VISTA is detected predominantly in CD68^+^ macrophages than CD3^+^ T cells and CD19^+^ B cells. **c** VISTA expressions in CD8^+^ cytotoxic cells were more than these in CD4^+^ helper-T lymphocytes

To visualize the expression landscape of VISTA in the TME of PDAC, we prepared TMAs from 137 cases of PDAC (cohort #1) using paraffin embedded tissues. With multiplex immunofluorescence, we studied the expression of multiple markers simultaneously in a single tissue section. By excluding samples with defoliation in the course of the experiment, and those with less than a 5% cancer compartment, we ultimately obtained 133 cases to study. We applied three different panels to demonstrate the levels of VISTA in tumor-infiltrating CD68^+^ macrophages, CD3^+^ T cells, CD19^+^ B cells, CD4^+^ T-helper cells, and CD8^+^ cytotoxic T cells, as well as to explore the association between VISTA expression and PD-1/PD-L1 (Supplementary Table S1 and Fig S2). VISTA was detected in all investigated IC subpopulations (Fig. 3a). Moreover, VISTA was expressed at significantly higher levels in CD68^+^ tumor-associated macrophages than in tumor-infiltrating CD3^+^ T or CD19^+^ B cells (Fig. 3b). Furthermore, the level of VISTA in CD8^+^ cytotoxic cells was higher than in CD4^+^ helper-T lymphocytes (Fig. 3c).

**Fig. 4:**
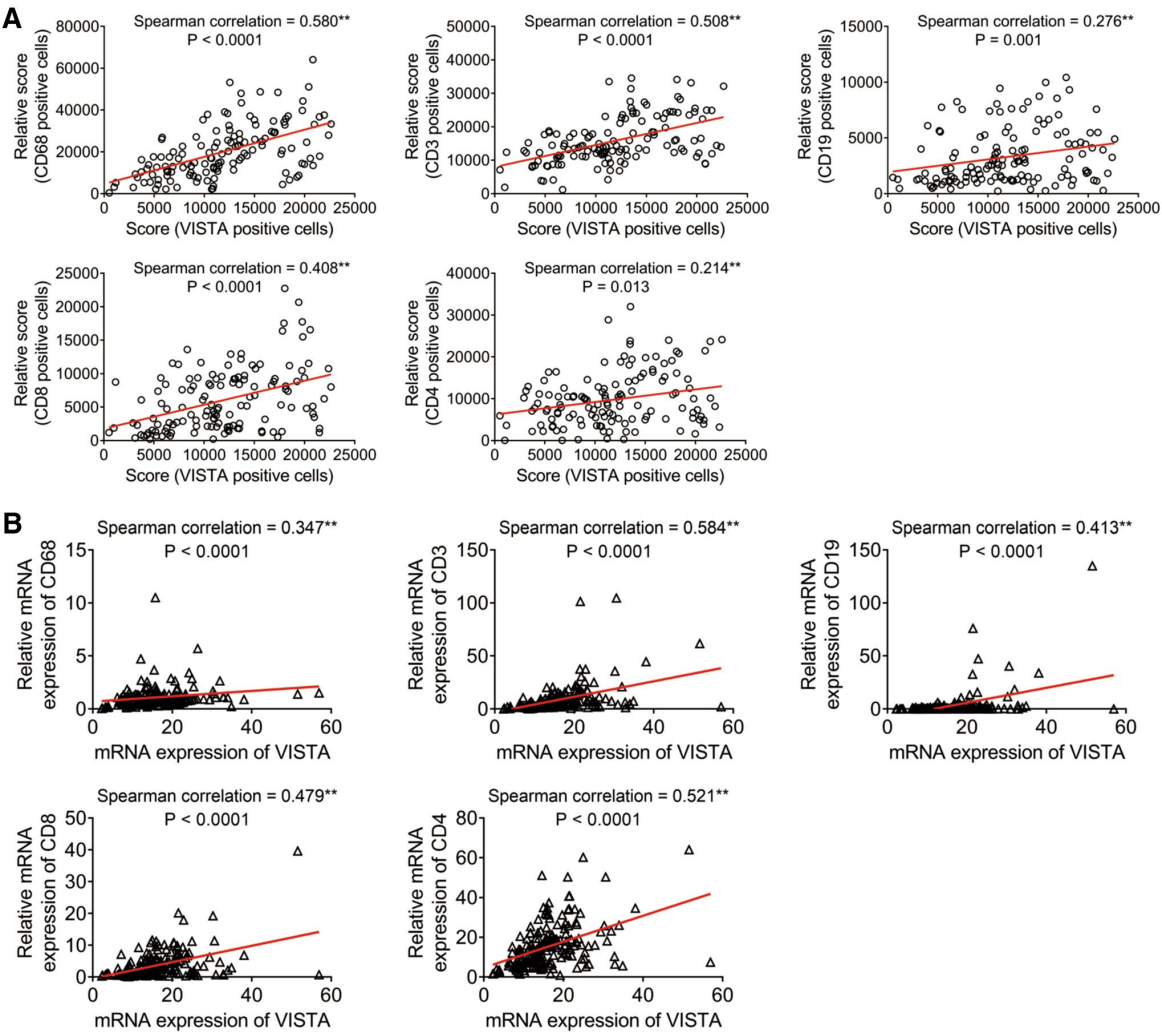
Correlation between VISTA levels and tumor infiltration of immune cell subpopulations in PDAC. **a** Positive correlation between VISTA level and tumor infiltration of CD68^+^ macrophages, CD3^+^ T cells, CD19^+^ B cells, CD8^+^ cytotoxic cells, or CD4^+^ helper-T lymphocytes in 137 individual human PDAC specimens. **b** Positive correlation between the expression of VISTA and CD68, CD3, CD19, CD8, or CD4 expression in 177 human PDAC samples from TCGA database

We also performed a correlation analysis to explore the relationship between VISTA expression and the infiltration levels of various immune cell subclusters. VISTA expression was positively related with tumor-infiltrating CD68^+^ macrophages, CD3^+^ T cells, CD19^+^ B cells, CD4^+^ T-helper cells, and CD8^+^ cytotoxic T cells (Fig. 4a). To validate our findings, we retrieved PDAC patient data from TCGA database. As shown in Fig. 4b, the mRNA expression levels of VISTA and CD3, CD4, CD8, CD19, and CD68 were positively correlated, which was consistent with our data. Altogether, these results indicated that the VISTA expression level was associated with infiltration of ICs within PDAC and played an important role in tumor immunology.

**Fig. 5:**
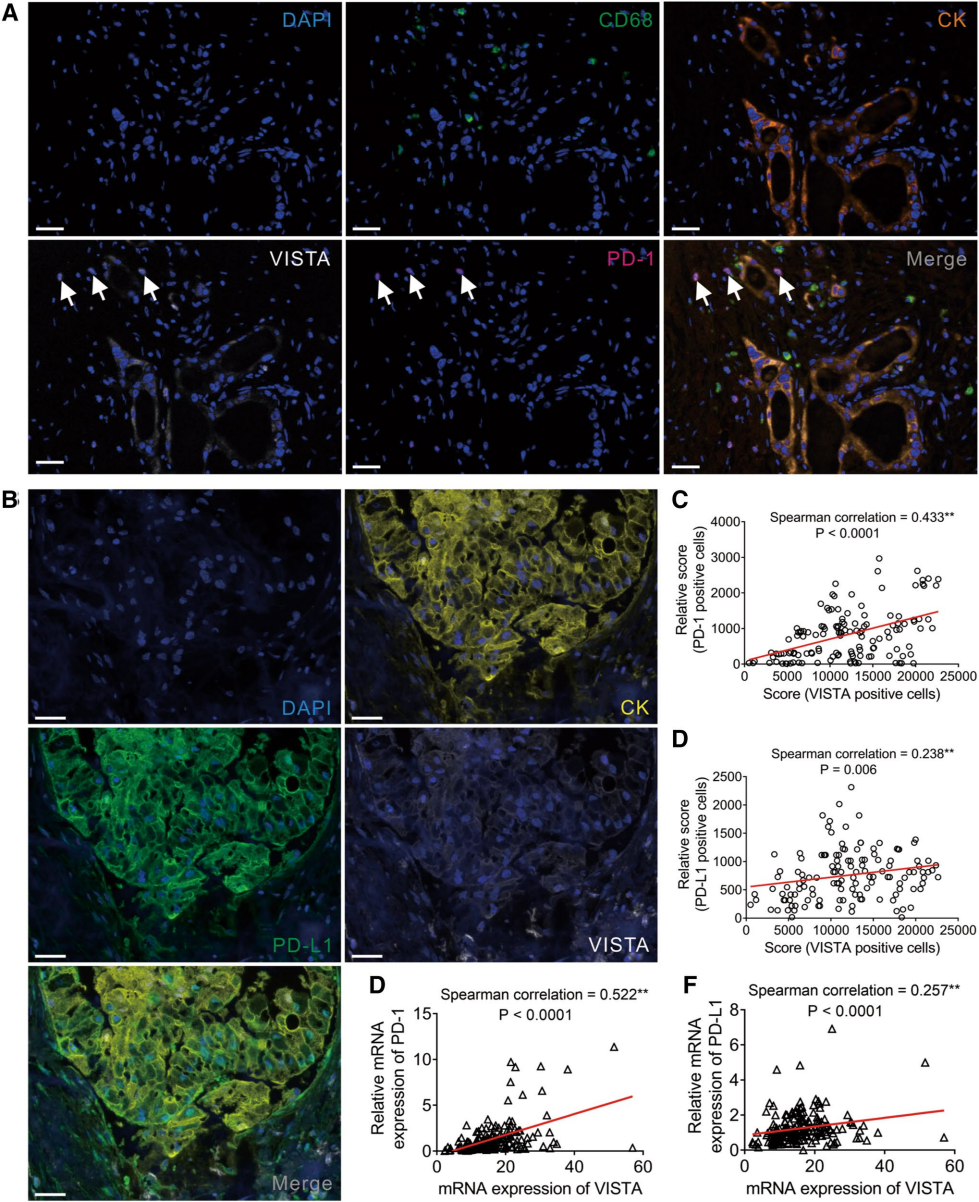
PD-1/PD-L1 expression is colocalized and correlated with VISTA expression in PDAC. **a** Representative colocalization of VISTA and PD-1 proteins in PDAC specimens with immune cells detection were shown. **b** Representative colocalization of VISTA and PD-L1 proteins in PDAC specimens with tumor cells detection were shown. **c**, **d** Positive correlation between the level of VISTA and PD-1 or PD-L1 level in 133 human PDAC specimens. **e**, **f** Positive correlation between the expression of VISTA and PD-1 or PD-L1 expression in 177 human PDAC samples from TCGA database. Scale bar = 100 μm (white line at the bottom left)

We then analyzed the relationship between VISTA and PD-1/PD-L1 expression of PDAC. Multiplex immunofluorescence revealed a co-localized expression pattern between VISTA and PD-1 or PD-L1 in the TME of PDAC (Fig. 5a, b). We then simultaneously measured and calculated the mean fluorescent intensities of VISTA, PD-1, and PD-L1 in the overall multispectral images to investigate the correlation of these variables. The expression of VISTA was positively correlated with the expression of both PD-1 and PD-L1 (*r* = 0.433, *p* < 0.001; *r* = 0.238, *p* = 0.006, respectively; Fig. 5c, d). To confirm the association between VISTA and PD-1/PD-L1 in PDAC, we assessed their mRNA expression levels using 177 PDAC cases from TCGA database. As shown in Fig. 5e, f, the expression of VISTA was positively correlated with PD-1 and PD-L1 expression (*r* = 0.522, *p* < 0.001; *r* = 0.26, *p* < 0.001, respectively). These results indicated that VISTA expression was spatially connected with PD-1/PD-L1 expression.

### The effect of anti-VISTA antibody treatment on liver metastasis of PDAC

**Fig. 6:**
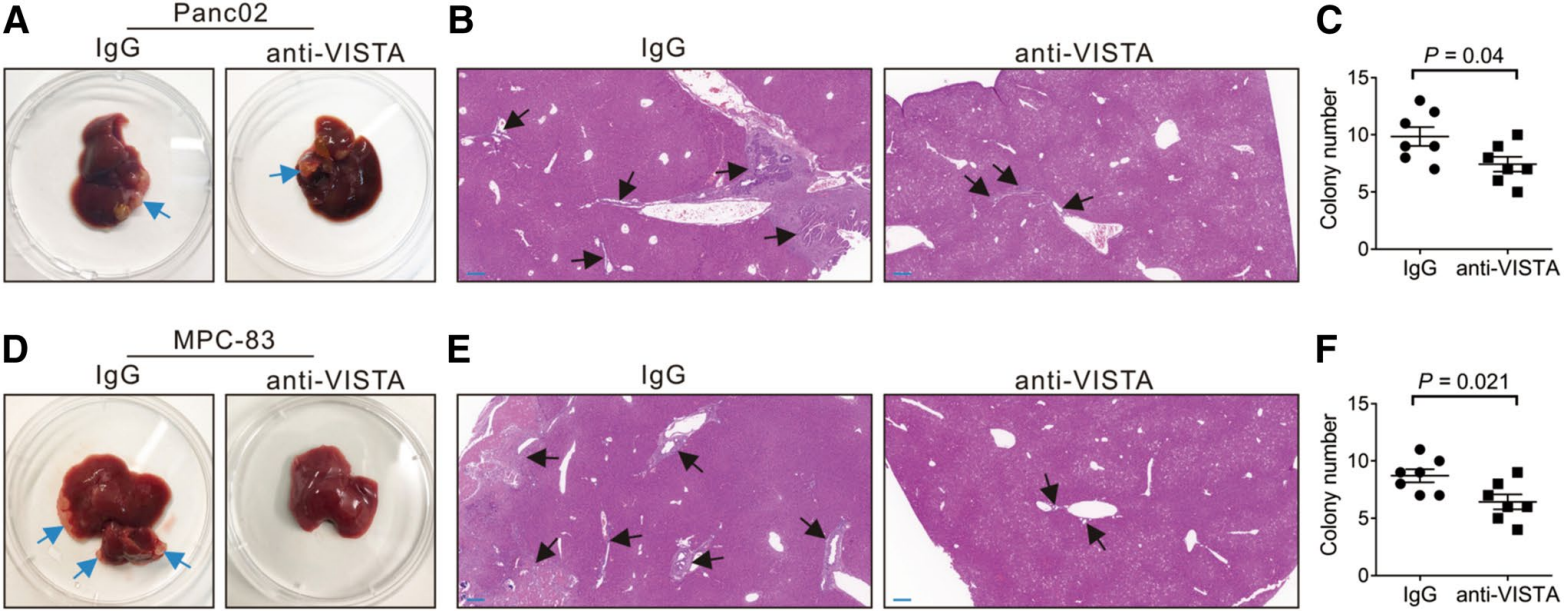
Anti-VISTA antibody inhibited tumor metastasis in mouse models Mice were treated with anti-VISTA antibody or mouse IgG. After 3-week treatment, mice were sacrificed and livers were harvested. Each group contained seven mice. **a** Representative livers from Panc02 mice were shown. Liver surface tumor nodules were indicated by blue arrows. **b** H&E staining was performed on sections of liver tissues of Panc02 mice. Metastatic tumor nodules outlined by the black arrows. Scale bar = 200 μm (blue line at the bottom left). **c** The number of metastatic tumors on histological sections of the midportion of the liver tissues of Panc02 mice were counted. **d–f** Representative livers from MPC-83 mice were shown, and the surface tumor nodules were indicated by blue arrows. H&E staining was performed on sections of liver tissues, and metastatic tumor nodules outlined by the black arrows. The number of metastatic tumors on histological sections of the midportion of the liver tissues were counted

Michaels et al. established models of pancreatic cancer liver metastasis in nude and NOD-scid-gamma mice by injecting human pancreatic cancer cells into the spleen. They found that hepatic macrophages could inhibit the progression of liver micrometastasis (Michaels et al. 2018). Moreover, studies have shown that abundant resident liver macrophages and infiltrating macrophages were subsistent in liver (Ciner et al. 2020; Gul et al. 2014; Paez et al. 2012; Racanelli and Rehermann 2006). In patients with pancreatic cancer, liver metastases can occur at an early stage, which seriously affects the prognosis. In this study, we found that VISTA was highly expressed in TAMs (Fig. 3a, b). A previous study showed that VISTA could suppress T cell proliferation and anti-VISTA antibody treatment significantly prolonged the survival of tumor-bearing mice (Mulati et al. 2019). To investigate the anti-tumor effects of an anti-VISTA antibody on liver metastases of pancreatic cancer, we injected Panc02 or MPC-83 cells into the spleen of C57BL/6 or KM mice, respectively, to establish mouse models of liver metastases of PDAC. Two weeks after injection of cancer cells, the mice were divided randomly into two groups for treatment with either an anti-VISTA mAb or a control mouse IgG mAb. Three weeks after treatment, the mice were euthanized and the livers were harvested. In both the Panc02 and MPC-83 tumor models, mice receiving the anti-VISTA treatment had significantly fewer metastatic nodules in the livers compared with mice treated with IgG (Fig. 6–f). These results suggest that an anti-VISTA antibody may be a potential treatment for liver metastases of PDAC.

## Discussion

VISTA is a novel immune checkpoint that is expressed predominantly within lymphoid organs and myeloid cells. It has been investigated in several tumor types. However, the role of VISTA in pancreatic cancer was unknown. Blando et al. (2019) examined tumor tissues from seven patients with pancreatic cancer and found that VISTA was expressed more frequently than PD-L1 in the tumor. Thus, VISTA may represent a new therapeutic target for pancreatic cancer. However, in their study, tumor tissue digested by enzymatic hydrolysis was used to detect VISTA expression by mass cytometry. That might alter the original characteristics of the cells and omit spatial information. In addition, the effect of anti-VISTA antibodies in PDAC has not been verified. In our study, we investigated the VISTA landscape by IHC and multiplex immunofluorescence comprehensively in large tissue sections from two different unrelated retrospective cohorts. Furthermore, we also preformed anti-VISTA antibody treatments in mouse models of pancreatic cancer with liver metastasis to verify the efficacy of this therapy in PDAC.

We found that VISTA was detectable in tumor-infiltrating CD68^+^ macrophages, CD3^+^ T cells, CD19^+^ B cells, CD4^+^ T-helper cells, and CD8^+^ cytotoxic T cells. Furthermore, VISTA was expressed at a significantly higher level in CD68^+^ tumor-associated macrophages. Previous studies demonstrated that the VISTA expressed in ICs could enforce the quiescence of T cells and function as a negative regulator (ElTanbouly et al. 2020; Wang et al. 2014). In addition, VISTA expression in antigen presenting cells restrained the activation of T cells, and VISTA in T cells acted as an inhibitory receptor (Ni and Dong 2017). Using a combination of single-cell RNA sequencing and single-cell ATAC sequencing technologies, ElTanbouly et al. demonstrated that genetic deletion of VISTA led to a reduction of the quiescent subset of T cells (ElTanbouly et al. 2020).

We found VISTA was also expressed in TCs and ECs of PDAC tumor tissues. In contrast, Liu et al. reported that VISTA was expressed primarily in ICs, and barely expressed in TCs of 52 PDAC tumor tissues (Liu et al. 2018). The different results about VISTA expression in TCs may be due to differences in sample sizes. Furthermore, we found that VISTA expression in TCs was significantly associated with longer overall survival. Most previous studies investigated the expression and function of VISTA in ICs, while the effect of VISTA in TCs remained unclear. Zong et al. and Zhang et al. reported that VISTA expressed in TCs was a favorable prognostic factor in patients with high-grade serous ovarian cancer and hepatoma (Zhang et al. 2018; Zong et al. 2020b). Those results were similar to our study. Overall, the function and mechanism of VISTA expression in TCs deserve further investigation.

Both VISTA and PD-1/PD-L1 are members of the B7 family and are homologous in molecular structure. VISTA was positively correlated with PD-L1 in gastric cancer, oral squamous cell carcinoma, and epithelioid malignant pleural mesothelioma (Boger et al. 2017; Muller et al. 2020; Wu et al. 2017). In addition, co-localization and positive correlation between VISTA and PD-1/PD-L1 at the protein level were found in non-small cell lung cancer (Villarroel-Espindola et al. 2018). A previous study demonstrated that VISTA and PD-1/PD-L1 could regulate T cell activation in tumors by different pathways (Liu et al. 2015). We found that VISTA was significantly associated and co-localized with PD-1/PD-L1 expression in PDAC. Our results also suggested that VISTA was positively associated with the level of T cell infiltration in the TME of PDAC, which is consistent with findings in hepatocellular carcinoma (Zhang et al. 2018). Taken together, these data suggest that VISTA and PD-1/PD-L1 may play a synergetic or cooperative role in PDAC immune evasion. However, further studies are urgently needed to explore the association between VISTA and PD-1/PD-L1 pathways in PDAC.

Immune checkpoint blockade represents an effective therapy in many advanced cancers. Recently, Mercier et al. demonstrated that VISTA mAbs could significantly suppress the growth of melanoma tumors in mouse models (Le Mercier et al. 2014). As well, Mulati et al. reported that an anti-VISTA antibody prolonged the survival of mice with ovarian tumors (Mulati et al. 2019). Anti-VISTA therapy might interrupt the immune escape process mediated by VISTA expressed in immune cells, resulting in tumor suppression. In the current study, we demonstrated that anti-VISTA antibody treatment could significantly reduce the number of metastatic tumor nodules in mouse models of PDAC with liver metastasis. A previous study reported that combination therapy with anti-VISTA and anti-PD-L1 antibodies achieved a synergistic therapeutic effect in a murine colon cancer model (Liu et al. 2015). Although the mechanism of anti-VISTA-mediated antitumor responses remains to be further investigated, combination therapy with anti-VISTA antibodies and other drugs may be a potential treatment strategy for PDAC.

In addition, our study had several limitations. In current study, we only investigated the profile of VISTA expression in the primary tumors and its relationship to prognosis, however, lacking the situation of VISTA expression in metastatic tumors. The profile of VISTA in metastases may be different from the primary tumors.

## Conclusion

In summary, the results of our study indicate that VISTA expression in TCs is an independent prognostic factor for PDAC patients, and expression of VISTA is associated with elevated tumor-infiltrating ICs and PD-1/PD-L1 expression. In addition, the use of anti-VISTA antibodies may be an effective therapeutic approach for PDAC.

## Electronic supplementary material

Below is the link to the electronic supplementary material.

Supplementary file1 (DOC 432 kb)
