# Supplementary material for: Prognostic significance and therapeutic potential of the immune checkpoint VISTA in pancreatic cancer
Source: J Cancer Res Clin Oncol. 2020 Nov 25;147(2):517–31. doi: 10.1007/s00432-020-03463-9 (PMC7817580; doi:10.1007/s00432-020-03463-9)
Supplement: Supplementary file 1 — Supplementary file1 (DOC 432 kb) [file 432_2020_3463_MOESM1_ESM.docx]

**Prognostic significance and therapeutic potential of the immune checkpoint VISTA in pancreatic cancer**

Zelin Hou^1#^, Yu Pan^1#^, Qinglin Fei^1, 2^, Yali Lin^1^, Yuanyuan Zhou^3^, Ying Liu^4^, Hongdan Guan^5^, Xunbin Yu^6^, Xianchao Lin^1^, Fengchun Lu^1^, Heguang Huang^1*^

**(Supplementary table S1-S4 and Supplementary figure S1-S2)**

**Supplementary table S1**

| Panel 1 | VISTA / PD-1H  Clone D5L5T, CST  Dilution 1:25, overnight, 4°C | CD68  Clone D4B9C, CST  Dilution 1:10000, 1 hour, RT | PD-L1  Clone EPR19759, Abcam  Dilution 1:250, overnight, 4°C | PD-1  Clone NAT105, Abcam  Dilution 1:25, overnight, 4°C | Pan-Cytokeratin  Clone PAN-CK (Cocktail), Abcam  Dilution 1:800, 1 hour, RT | Nuclei staining  DAPI, 1µg/ml, 5 min, RT |
| --- | --- | --- | --- | --- | --- | --- |
| Panel 2 | VISTA / PD-1H  Clone D5L5T, CST  Dilution 1:25, overnight, 4°C | CD68  Clone D4B9C, CST  Dilution 1:10000, 1 hour, RT | CD4  Clone EPR6855, Abcam  Dilution 1:100, 1 hour, RT | CD8  Clone EP1150Y, Abcam  Dilution 1:500, 1 hour, RT | Pan-Cytokeratin  Clone PAN-CK (Cocktail), Abcam  Dilution 1:800, 1 hour, RT | Nuclei staining  DAPI, 1µg/ml, 5 min, RT |
| Panel 3 | VISTA / PD-1H  Clone D5L5T, CST  Dilution 1:25, overnight, 4°C | CD68  Clone D4B9C, CST  Dilution 1:10000, 1 hour, RT | CD3  Clone PS1, Abcam  Dilution 1:100, 1 hour, RT | CD19  Clone EPR5906, Abcam  Dilution 1:500, 1 hour, RT | Pan-Cytokeratin  Clone PAN-CK (Cocktail), Abcam  Dilution 1:800, 1 hour, RT | Nuclei staining  DAPI, 1µg/ml, 5 min, RT |
| Opal staining | Opal 520 | Opal 540 | Opal 570 | Opal 620 | Opal 650 | DAPI |

**Table S2. Clinic-pathological characteristics**

| **Cohort description** | **Cohort #1** | **Cohort #2** |
| --- | --- | --- |
| **Total patients** | 137 | 86 |
| **Gender** |  |  |
| **Male** | 88 | 55 |
| **Female** | 49 | 31 |
| **Age (years)** |  |  |
| **≥ 65** | 57 | 36 |
| **< 65** | 80 | 50 |
| **Localization** |  |  |
| **Head** | 88 | 54 |
| **Other** | 49 | 32 |
| **Grading** |  |  |
| **G1** | 6 | 3 |
| **G2** | 102 | 51 |
| **G3** | 28 | 29 |
| **G4** | 1 | 3 |
| **pT-staging** |  |  |
| **pT1** | 4 | 3 |
| **pT2** | 48 | 29 |
| **pT3** | 57 | 39 |
| **pT4** | 28 | 15 |
| **pN-staging** |  |  |
| **pN0** | 56 | 30 |
| **pN1** | 54 | 30 |
| **pN2** | 27 | 26 |
| **pStage** |  |  |
| **Ⅰ** | 21 | 11 |
| **Ⅱ** | 63 | 38 |
| **Ⅲ** | 45 | 30 |
| **Ⅳ** | 8 | 7 |
| **Vascular invasion** | |  |
| **Yes** | 37 | 25 |
| **No** | 100 | 61 |
| **Diameter (cm)** |  |  |
| **≥ 4** | 69 | 26 |
| **< 4** | 68 | 60 |
| **Procedure** |  |  |
| **PD** | 85 | 50 |
| **DP** | 43 | 27 |
| **TP** | 9 | 9 |
| **Postoperative chemotherapy** | |  |
| **Yes** | 57 | 39 |
| **No** | 80 | 47 |

| **Table S3.** VISTA expression in PDAC tissues | | | | | | | | |
| --- | --- | --- | --- | --- | --- | --- | --- | --- |
|  | **TCs** | |  | **ICs** | |  | **ECs** | |
| **Cohort** | **Low** | **High** |  | **Low** | **High** |  | **Low** | **High** |
| Cohort #1 (N=137) | 101(73.72%) | 36(26.28%) |  | 82(59.85%) | 55(40.15%) |  | 105(76.64%) | 32(23.36%) |
| Cohort #1 (N=86) | 65(75.58%) | 21(24.42%) |  | 56(65.12%) | 30(34.88%) |  | 60(69.77%) | 26(30.23%) |
| **Abbreviations:** TC, tumor cells; IC, immune cells; ECs, endothelial cells | | | | | | | | |

| **Table S4.** Spearman rank correlation analysis | | | | | | | | | |
| --- | --- | --- | --- | --- | --- | --- | --- | --- | --- |
|  |  | **VISTA in TCs** | |  | **VISTA in ICs** | |  | **VISTA in ECs** | |
|  |  | **r** | **P** |  | **r** | **P** |  | **r** | **P** |
| **Cohort #1 (N=137)** | VISTA in TCs | 1 | **-** |  | 0.12 | 0.162 |  | 0.219 | **0.01** |
|  | VISTA in ICs | 0.12 | 0.162 |  | 1 | - |  | 0.076 | 0.379 |
|  | VISTA in ECs | 0.219 | **0.01** |  | 0.076 | 0.379 |  | 1 | - |
| **Cohort #2 (N=86)** | VISTA in TCs | 1 | - |  | 0.095 | 0.384 |  | 0.333 | **0.002** |
|  | VISTA in ICs | 0.095 | 0.384 |  | 1 | - |  | -0.057 | 0.603 |
|  | VISTA in ECs | 0.333 | **0.002** |  | -0.057 | 0.603 |  | 1 | - |
| **Abbreviations:** TC, tumor cells; IC, immune cells; ECs, endothelial cells | | | | | | | | | |


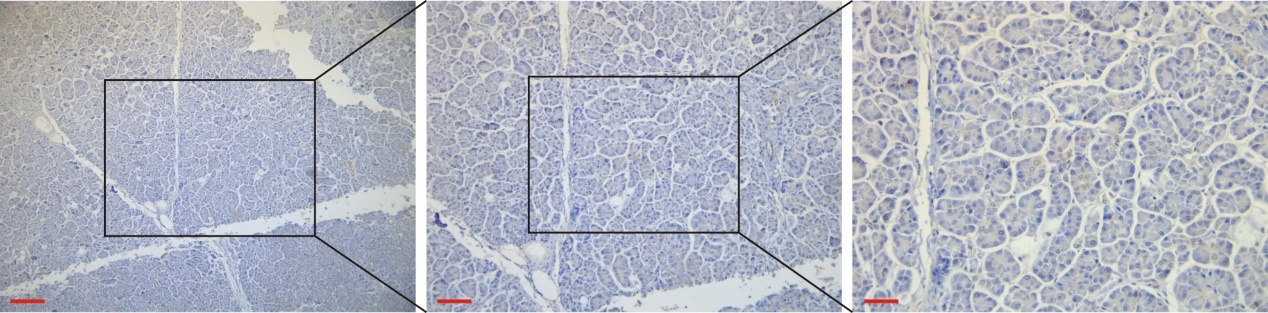


**Figure S1.** Expression of VISTA in adjacent normal tissues of PDAC patients. Staining with an anti-VISTA in the human PDAC adjacent normal tissue samples. Scale bar = 100 µm (red line at the bottom left)


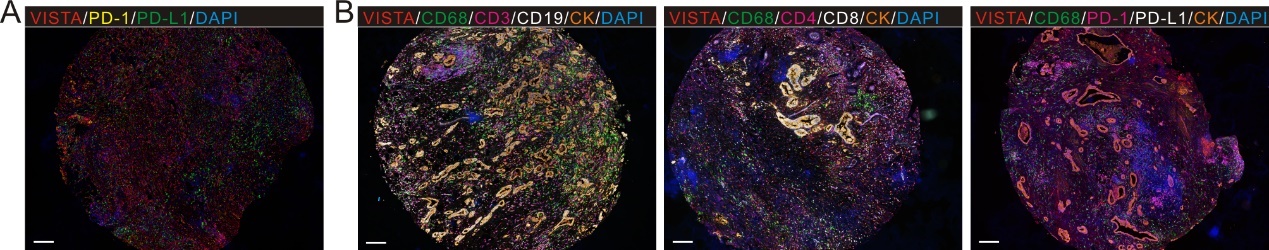


**Figure S2.** The staining panels of multiplex immunofluorescence**.** (**A**) Multiplex immunofluorescence staining image of tonsil control tissue. Scale bars =100 μm. (**B**) Three different panels of multiplex immunofluorescence in PDAC tumor microenvironment. Scale bars =100 μm.
